# Correction: Assessing independence in mobility activities in trauma care: Validity and reliability of the Activity Independence Measure-Trauma (AIM-T) in humanitarian settings

**DOI:** 10.1371/journal.pgph.0002953

**Published:** 2024-02-28

**Authors:** Bérangère Gohy, Christina H. Opava, Johan von Schreeb, Rafael Van den Bergh, Aude Brus, Nicole Fouda Mbarga, Jean Patrick Ouamba, Jean-Marie Mafuko, Irene Mulombwe Musambi, Delphine Rougeon, Evelyne Côté Grenier, Lívia Gaspar Fernandes, Julie Van Hulse, Eric Weerts, The AIM-T Study Group, Nina Brodin

There are errors in the Funding and Competing Interests statements. The correct statements are:

**Funding:** Elrha’s Research for Health in Humanitarian Crises (R2HC) programme (Grant No.32398) funded the research coordination (BG) and research activity for this study, funded by the UK Foreign, Commonwealth and Development Office (FCDO), Wellcome, and the Department of Health and Social Care (DHSC) through the National Institute for Health Research (NIHR) (https://www.elrha.org/programme/research-for-health-in-humanitarian-crises/). Médecins Sans Frontières (MSF) provided support in the form of salaries for RVdB, NFM, JPO, JMM, IMM, DR, ECG, LF, JVH, and for AIM-T study group members GFT, EN, IC, AA, and AM. The specific roles of these authors are articulated in the ‘author contributions’ section. The routine care for patients was covered by MSF’s programmatic funding. The funders otherwise had no role in the study design, data collection and analysis, decision to publish, or preparation of the manuscript.

**Competing Interests:** The authors have read the journal’s policy and have the following competing interests to declare: Médecins Sans Frontières (MSF) provided support in the form of salaries for RVdB, NFM, JPO, JMM, IMM, DR, ECG, LF, JVH, and for AIM-T study group members GFT, EN, IC, AA, and AM. This does not alter our adherence to *PLOS Global Public Health* policies on sharing data and materials. There are no patents, products in development, or marketed products associated with this research to declare.
