# Supplementary material for: Assessing independence in mobility activities in trauma care: Validity and reliability of the Activity Independence Measure-Trauma (AIM-T) in humanitarian settings
Source: PLOS Glob Public Health. 2023 Sep 11;3(9):e0001723. doi: 10.1371/journal.pgph.0001723 (PMC10495016; doi:10.1371/journal.pgph.0001723)
Supplement: S1 Fig — (PDF) [file pgph.0001723.s001.pdf]

S1 Fig Activity Independence Measure – Trauma (AIM-T)

ACTIVITY INDEPENDENCE MEASURE-TRAUMA (AIM-T)

THE MEASURE

CORE

Changing and maintaining body position						
Roll over	0	1	2	3	4	5
Sit up and remain seated for 10 sec.	0	1	2	3	4	5
**TOTAL Core subscale**	___ / 10					

LOWER LIMB

Changing and maintaining body position						
Stand up and remain standing for 10sec.	0	1	2	3	4	5
Kneel down and stand up	0	1	2	3	4	5
**Walking and moving**						
Walk/Move around 14m	0	1	2	3	4	5
Timed 10m walk/move around *	0	1	2	3	4	5
Climb up and down 10 steps	0	1	2	3	4	5
**TOTAL Lower Limb subscale**	___ / 25					

UPPER LIMB

Fine hand use						
Pick up a small object and manipulate	0	1	2	3	4	5
**Hand and arm use**						
Open a jar/bottle	0	1	2	3	4	5
Reach lower back and grasp clothes	0	1	2	3	4	5
Reach face and neck	0	1	2	3	4	5
**Lifting and carrying objects**						
Lift and Carry 5kg above shoulder level	0	1	2	3	4	5
**TOTAL Upper Limb subscale**	___ / 25					

0 = total dependence; 1 = need for human assistance; 2 = need for material assistance, with difficulties; 3 = need for material assistance, without difficulties; 4 = independence with difficulties; 5 = total independence

* For timed 10m walk/move: **if >12.5 seconds:** 0= total dependence; 1 = need for human assistance; **if ≤12.5 seconds:** 2 = need for material assistance, with difficulties; 3 = need for material assistance, without difficulties; 4 = independence with difficulties; 5 = total independence

CC BY
