# Supplementary material for: Assessing independence in mobility activities in trauma care: Validity and reliability of the Activity Independence Measure-Trauma (AIM-T) in humanitarian settings
Source: PLOS Glob Public Health. 2023 Sep 11;3(9):e0001723. doi: 10.1371/journal.pgph.0001723 (PMC10495016; doi:10.1371/journal.pgph.0001723)

S2 Fig. The three-factor model of the AIM-T with factor loadings. The three subscales are the latent constructs, depicted by circles, while each of the AIM-T activities are the items, depicted in rectangles. The straight lines show which items loads on which latent construct, with factor loadings indicated accordingly.

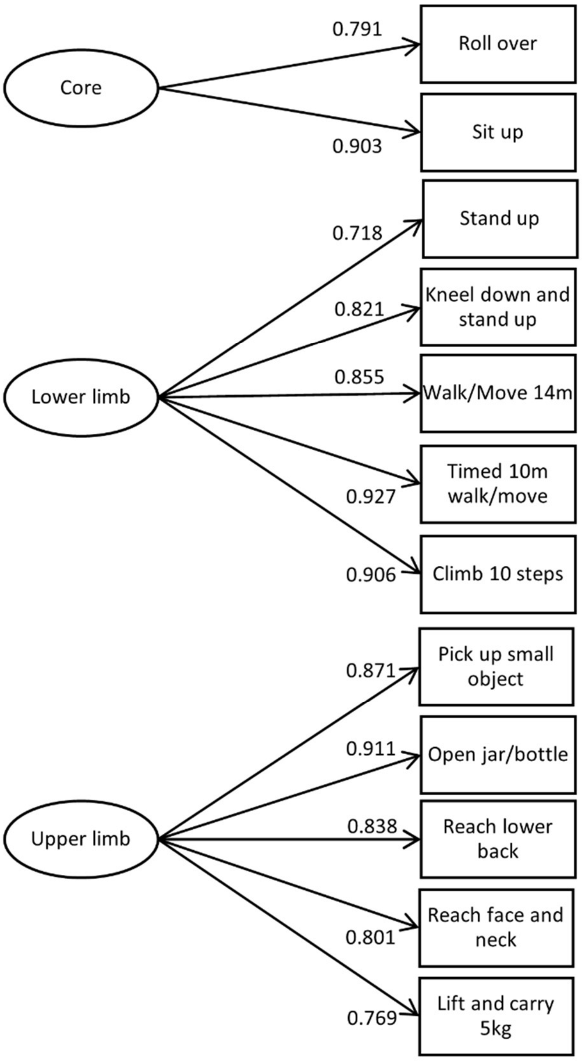

Supplement: S2 Fig — (PDF) [file pgph.0001723.s002.pdf]
