# Supplementary material for: Assessing independence in mobility activities in trauma care: Validity and reliability of the Activity Independence Measure-Trauma (AIM-T) in humanitarian settings
Source: PLOS Glob Public Health. 2023 Sep 11;3(9):e0001723. doi: 10.1371/journal.pgph.0001723 (PMC10495016; doi:10.1371/journal.pgph.0001723)

S3 Fig. Bland and Altman plots for the AIM-T subscales

S3A Fig. Bland and Altman plot of the differences in AIM-T core subscale scores between the first and the second raters against the pooled mean of the AIM-T core subscale scores for patients after trauma in four humanitarian settings (n=77). The solid line represents the mean difference between the two raters (0.18), while the dashed lines represent the limits of agreement (-2.46 to 2.82).

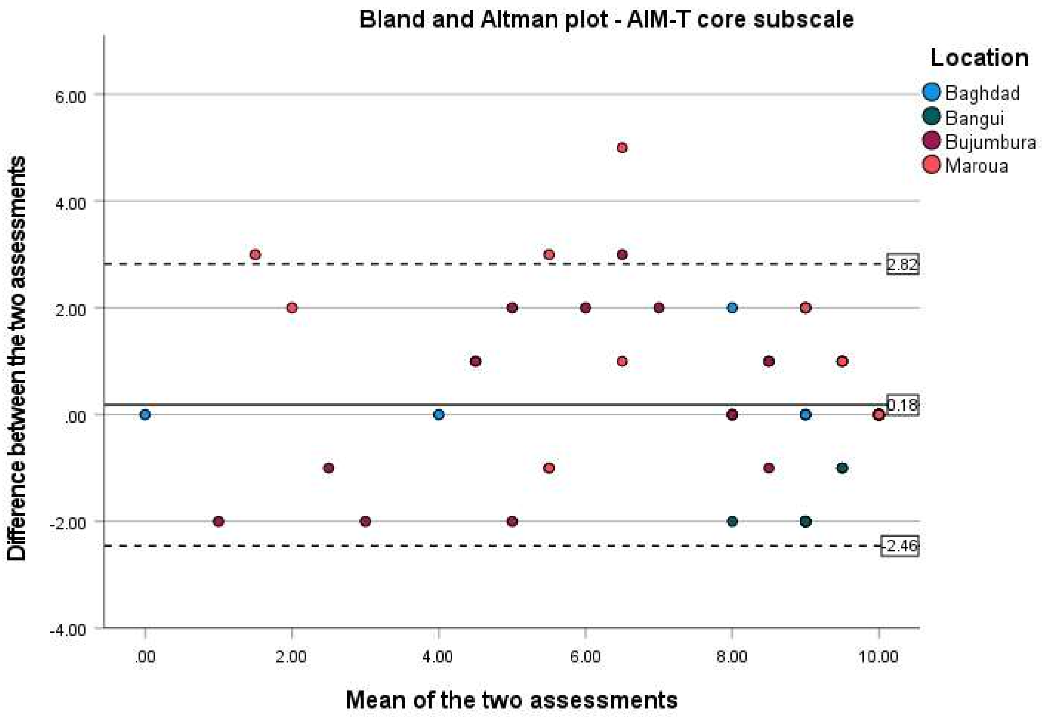

S3B Fig. Bland and Altman plot of the differences in AIM-T lower limb subscale scores between the first and the second raters against the pooled mean of the AIM-T lower limb subscale scores for patients after trauma in four humanitarian settings (n=77). The solid line represents the mean difference between the two raters (0.69), while the dashed lines represent the limits of agreement (-5.89 to 7.27).

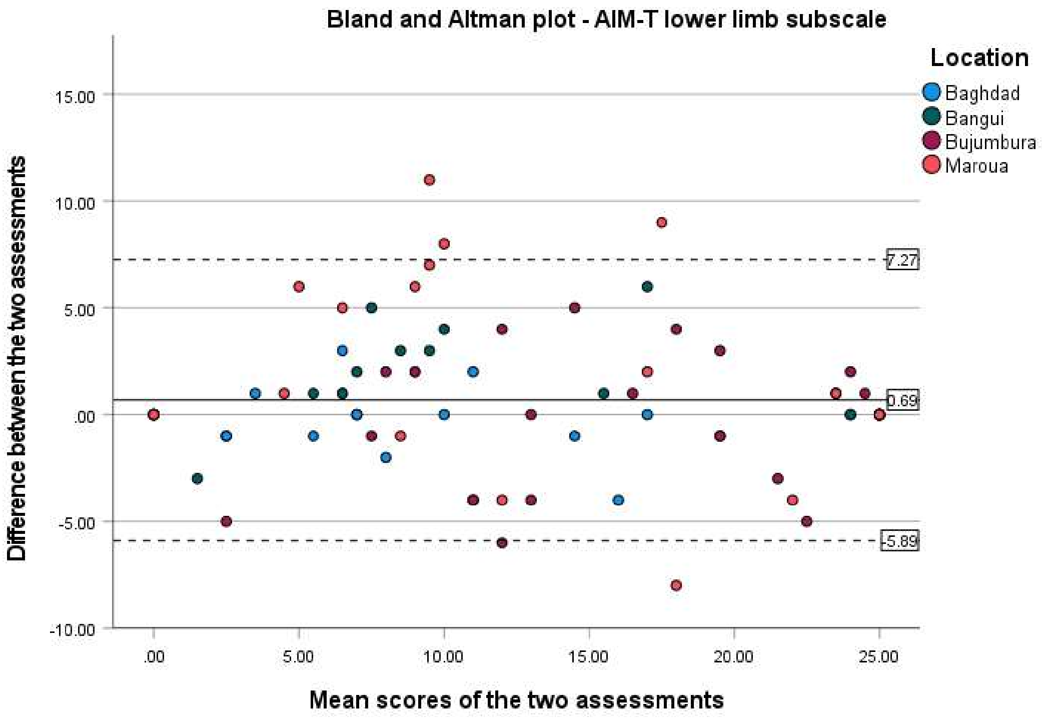

S3C Fig. Bland and Altman plot of the differences in AIM-T upper limb subscale scores between the first and the second raters against the pooled mean of the AIM-T upper limb subscale scores for patients after trauma in four humanitarian settings (n=77). The solid line represents the mean difference between the two raters (0.43), while the dashed lines represent the limits of agreement (-4.67 to 5.6).

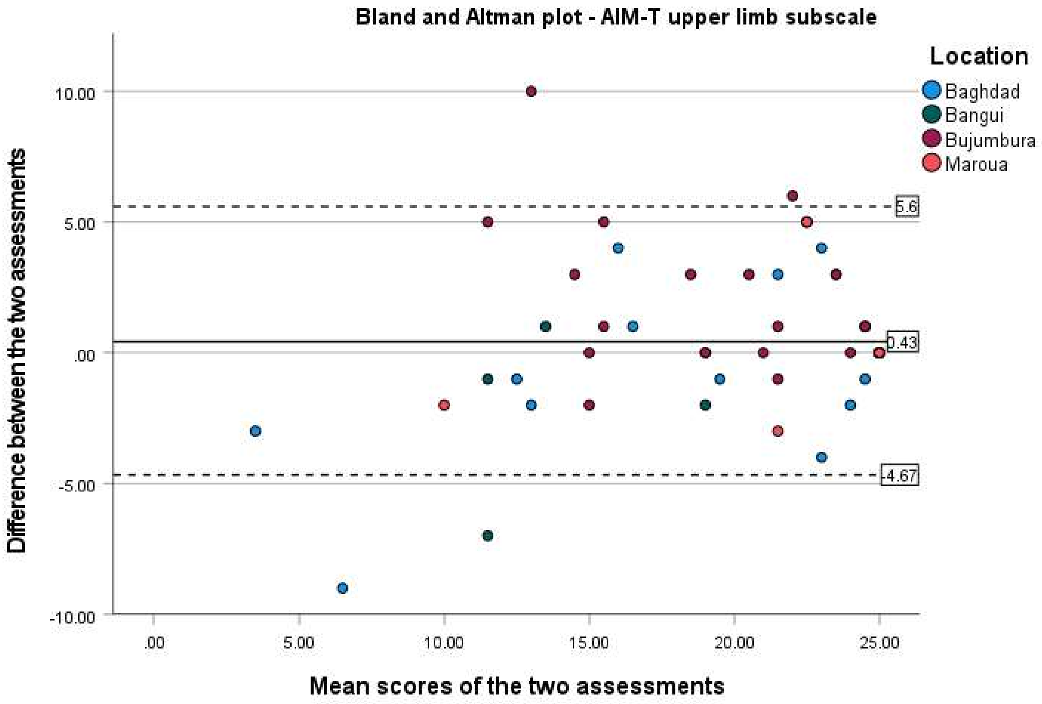

Supplement: S3 Fig — (PDF) [file pgph.0001723.s003.pdf]
