# Supplementary material for: Assessing independence in mobility activities in trauma care: Validity and reliability of the Activity Independence Measure-Trauma (AIM-T) in humanitarian settings
Source: PLOS Glob Public Health. 2023 Sep 11;3(9):e0001723. doi: 10.1371/journal.pgph.0001723 (PMC10495016; doi:10.1371/journal.pgph.0001723)
Supplement: S4 Fig — (PDF) [file pgph.0001723.s004.pdf]

S4 Fig The Activity Independence Measure – Trauma (AIM-T) poster and scoring system flowchart

ACTIVITY INDEPENDENCE MEASURE-TRAUMA (AIM-T)

12 ACTIVITIES

CORE

1 ROLL OVER

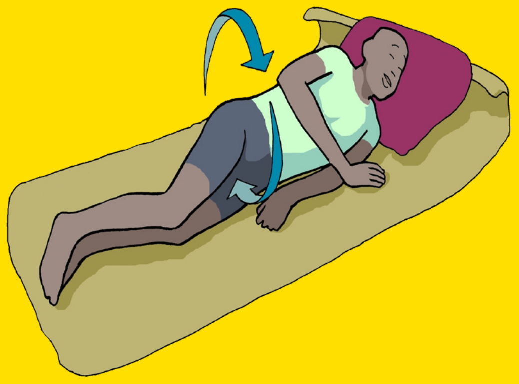

2 SIT UP AND REMAIN SEATED FOR 10 SEC.

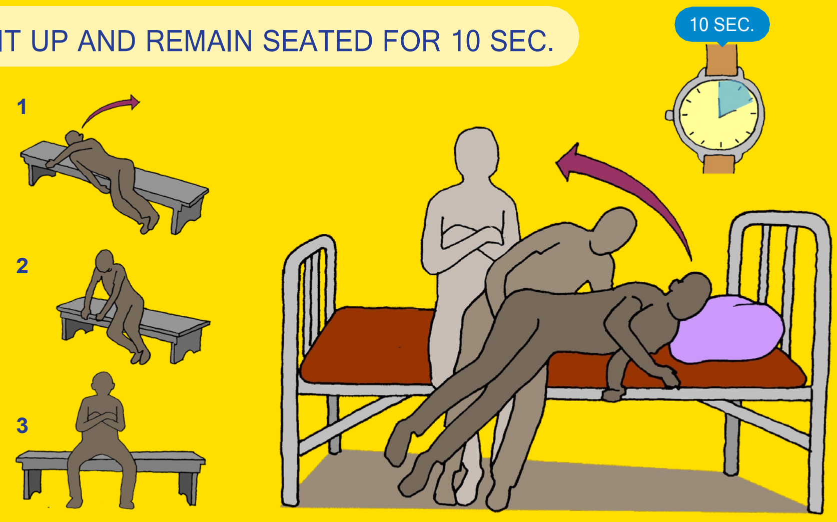

LOWER LIMB

3 STAND UP AND REMAIN STANDING FOR 10 SEC.

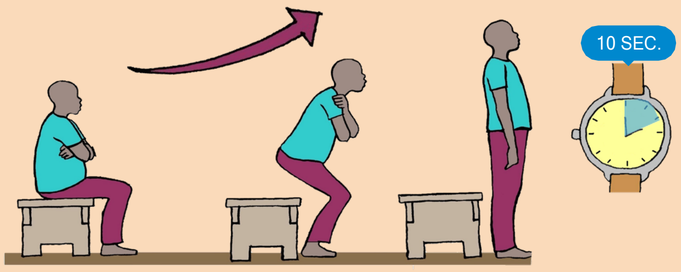

4 WALK/MOVE AROUND 14 METERS

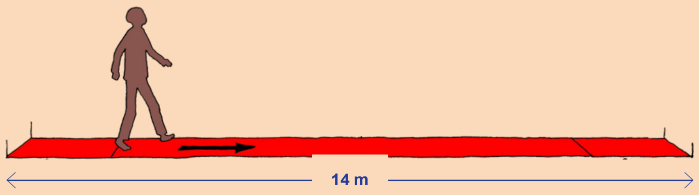

5 TIMED 10 METER WALK/MOVE AROUND

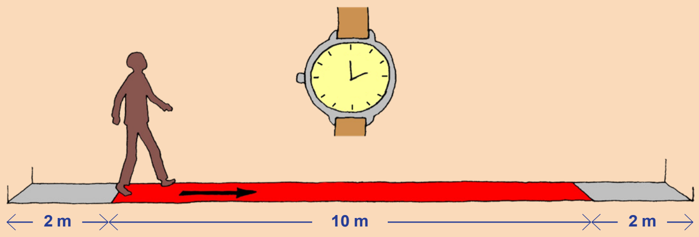

6 CLIMB UP AND DOWN 10 STEPS

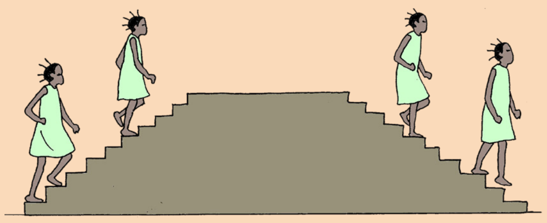

7 KNEEL DOWN AND STAND UP

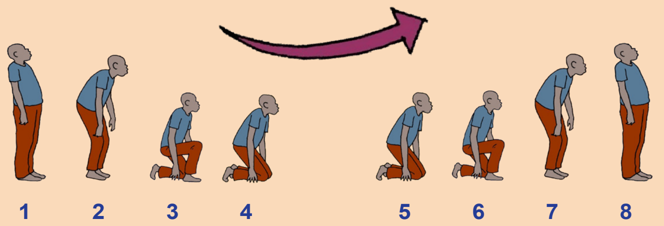

UPPER LIMB

8 PICK UP A SMALL OBJECT AND MANIPULATE

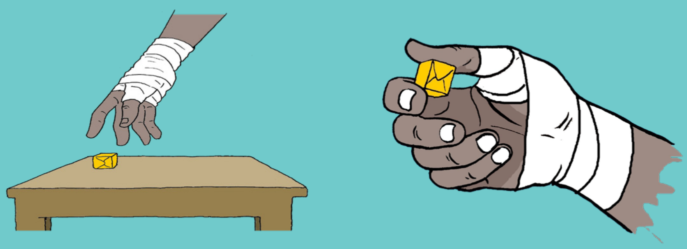

9 OPEN A JAR/BOTTLE

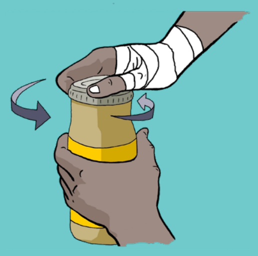

10 REACH LOWER BACK AND GRASP CLOTHES

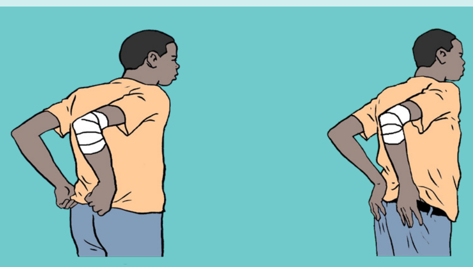

11 REACHING FACE AND NECK

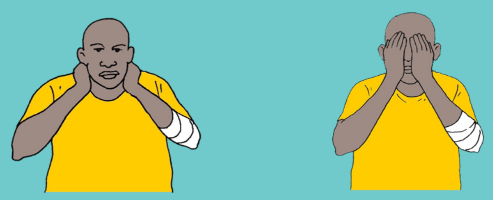

12 LIFT AND CARRY 5KG ABOVE SHOULDER LEVEL

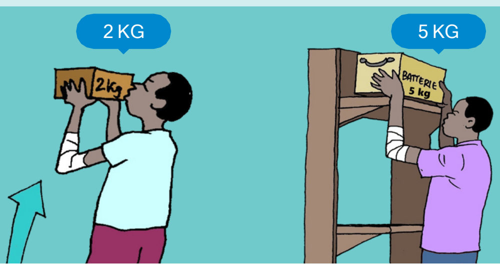

CC BY

Activity Independence Measure – Trauma (AIM-T) scoring system

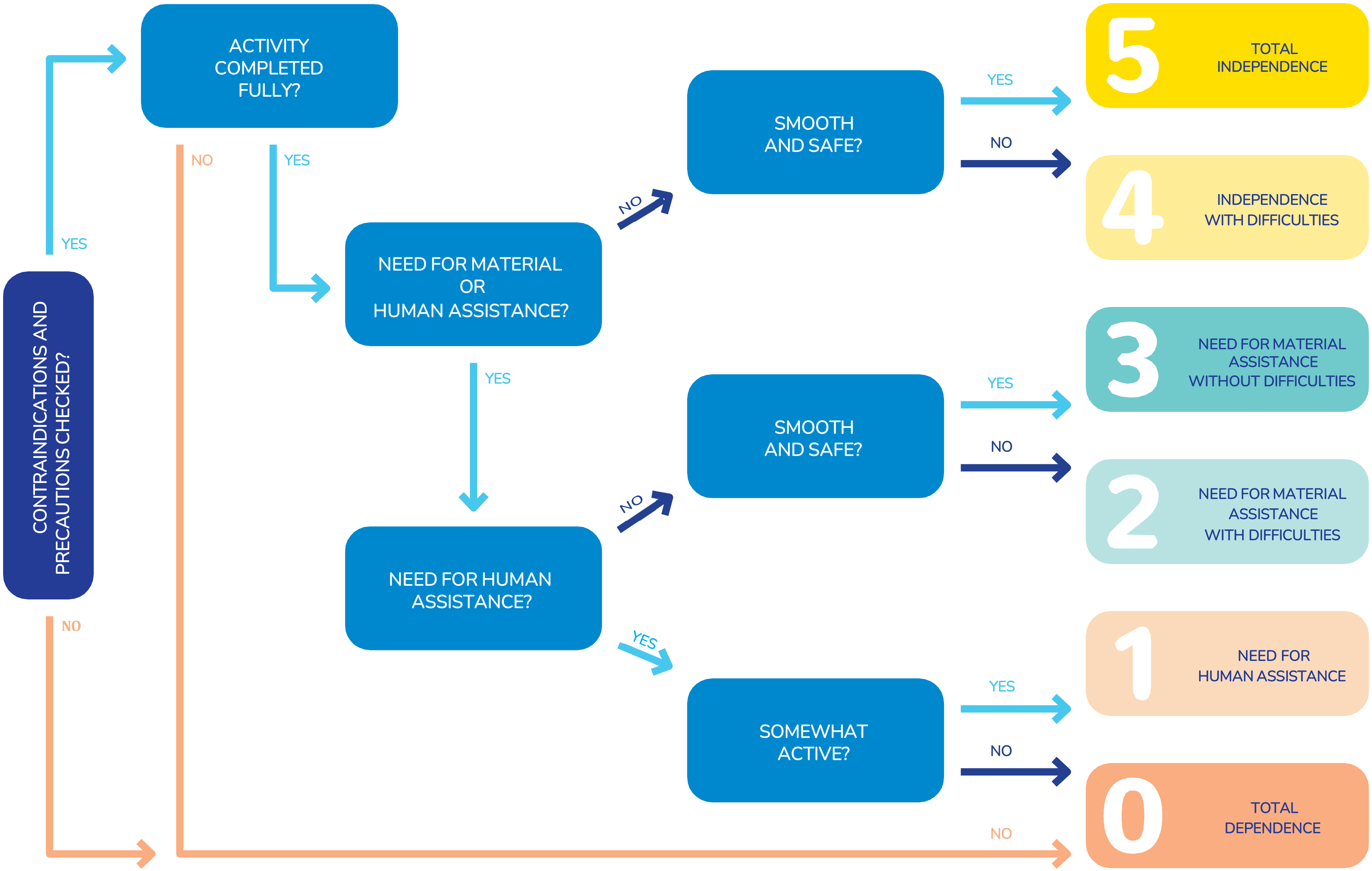

CC BY
