# Supplementary material for: Assessing independence in mobility activities in trauma care: Validity and reliability of the Activity Independence Measure-Trauma (AIM-T) in humanitarian settings
Source: PLOS Glob Public Health. 2023 Sep 11;3(9):e0001723. doi: 10.1371/journal.pgph.0001723 (PMC10495016; doi:10.1371/journal.pgph.0001723)
Supplement: Attachment — Submitted filename: Response to reviewers.docx [file pgph.0001723.s005.docx]

Dear Editor and reviewers,

Please find below a point-by-point response to your comments, as well as changes made accordingly.

Best regards,

Berangere Gohy

|  | Response to point raised | Change(s) made |
| --- | --- | --- |
| 1. We noticed you have some minor occurrence of overlapping text with the following previous publication(s), which needs to be addressed:  - <https://doi.org/10.1371/journal.pgph.0001334> In your revision ensure you cite all your sources (including your own works), and quote or rephrase any duplicated text outside the methods section. Further consideration is dependent on these concerns being addressed." | Thank you for identifying this overlapping text. | We have now quoted and rephrased the overlapping text. |
| Please review your reference list to ensure that it is complete and correct. If you have cited papers that have been retracted, please include the rationale for doing so in the manuscript text, or remove these references and replace them with relevant current references. Any changes to the reference list should be mentioned in the rebuttal letter that accompanies your revised manuscript. If you need to cite a retracted article, indicate the article’s retracted status in the References list and also include a citation and full reference for the retraction notice. | Thank you. | We have now reviewed all references and edited when required. |
| - Introduction: clearly written, appropriately contextualizes the gap addressed by the authors manuscript (e.g. importance of a functional disability metric appropriate to humanitarian settings). They do an excellent job of highlighting the limitations of existing scores (e.g. cannot be implemented in settings of limited literacy, overly lengthy, culturally inappropriate.) **The only change I would recommend is that, as written, it is slightly unclear whether this is the first time the AIM-T is being presented as a score, or whether the AIM-T has previously been described and the authors here are conducting a validity assessment.** Would recommend they provide clarification. | Thank you for your suggestion. The AIM-T has indeed been first described in a scientific publication in 2016, before being revised based on item reduction and content validity, as published in 2022. | We have now rephrased and clarified. |
| - General: minor proofreading is required throughout the manuscript (e.g. “…centres supported or ran by…” should read “centres supported or run by,” page 6) | Thank you for identifying this typo. | We have proofread the full manuscript and have corrected errors where identified. |
| - Methods: well organized and well presented. Although this is minor, the authors select a very odd and obscure reference to discuss their use of the SATS as a triage score. They should (a) cite this the first time it is used (currently the reference is appended to the second sentence) and (b) select a more relevant reference regarding the SATS. | Thank you for your comment.  The reference had been chosen as it refers to the SATS use in a context similar to the study centres.  The initial publication on the SATS we identified is referring to it with its first name (i.e. Cape Triage Score), we have therefore also added the SATS manual. | We have now moved the citation to the correct place and have also added two original references, i.e. the first publication describing the triage score (named Cape Triage Score at that time), as well as the SATS manual. |
| Only other additional comment is that the methods section seems unnecessarily long. The authors could consider making this more concise, particularly the section describing the various measurements scores and procedures which contain excessive detail that makes the manuscript unwieldy. | Thank you for your comment. We have now shortened the measurement and procedure sections as much as possible. However, based on the second reviewer’s comments, we have also included some additional information, such as training languages. | The two sections have been revised. |
| Results: age of raters presented in the demographics section does not seem relevant | Thank you for your comment. | We have removed both sex and age of raters. |
| - Discussion: Overall this is the section that requires the most reconsideration. On page 20, recommend the authors reword this paragraph. As written, it is unclear what they propose the AIM-T to offer over the BI after they raise the point that added value is equivocal. This sentence is confusing and unclear as written: “Thus, by including only culturally acceptable activities, the AIM-T aims to be composed of activities performed similarly across settings, thereby promoting early mobilization while also facilitating comparability of results across settings..” | Thank you for the feedback: the two main advantages of the AIM-T concern the inclusion of activities performed similarly across patients and their appropriateness for observation. | This paragraph has now been rephrased around the two mentioned main advantages. |
| Recommend the authors restructure the final paragraphs of the section. It is unconventional to have a section “strengths and limitations” at the end of the discussion as opposed to just limitations. The discussion would be more compelling and coherent if contexualization of its strengths were integrated with the discussion of its validity/reliability, and just a short paragraph on limitations prior to conclusion were presented in the standard fashion.  The authors should also restructure the discussion section such that “implications and future research” is presented prior to the limitations paragraph | Thank you for your suggestion. However, we have aimed to maintain the structure of our previous paper on the AIM-T (PLOS GPH, December 2022), and propose to keep the sections as written, in order to keep the homogeneity between these papers. | The strengths, limitations, and implications and future research paragraphs have not been restructured. |
|  |  |  |
| - Conclusion: should contain a sentence clearly stating what the AIM-T adds to prior research | Thank you for your suggestion. | We have now related this study with recent recommendations from the WHA resolution on rehabilitation, and have added a sentence to conclude the manuscript |
| Reviewer #2 |  |  |
| Page 4: the link between ‘burden of injury’ and ‘people living with disability’ is not clear. Both groups are not necessarily the same, especially if the authors write ‘persons living with disability’ rather than something like ‘persons with trauma-caused disability’. A sentence explaining the link between injury and resulting impairment or certain forms of disability would help. | Thank you for identifying this need for clarification. Indeed, not all persons living with disability have had an injury, but disability can result from injury. Moreover, regardless of the health condition related to disability, barriers to access essential services are faced in humanitarian settings. We want to ensure that these elements remain central in our reasoning. | We have now specified that disability can result from injury, as well as from other health conditions. |
| - Page 5: lack of language validation of outcome measures is another barrier in addition to lack of cultural validation | Thank you for pointing out this additional barrier.  The lack of availability in the target languages is indeed hindering use of common (patient-reported) measures, which have been recently reviewed by VanZyl et al. across languages spoken in sub-Saharan Africa (Van Zyl et al. 2023). | We have now added this aspect, together with a new reference. |
| - Page 5: “for clinicians to mobilize patients earlier” – it may help to define which professional groups are supposed to apply the measure | The AIM-T is designed to be provided by any trained healthcare professionals involved in trauma care. | “Clinicians” has been changed to “healthcare professionals”, and have also specified in the “clinical implications” section that “any” healthcare professionals could use the AIM-T. |
| - “It was purposively developed as an observed measure to encourage patients to move early on in their care process and for clinicians to mobilize patients earlier, both of which are known challenges in humanitarian settings” – this sentence seems a bit out of place; outcome measurement development and changing patient/clinician behaviour around early mobilization are two different concepts; outcomes measures should be developed to measure outcomes, see also comment below re discussion; | While these are indeed two different concepts, we find that - in particular in humanitarian contexts - having an observed measure provides opportunities to make the patient move, both directly by performing the activities contained in the measure, and indirectly by emphasizing the importance of early mobilization due to its inclusion in the measurement process. | This paragraph has been reworked. |
| - Page 7: participate in the study (not ‘to’) | Thank you for identifying this typo. | This typo was corrected. |
| - Page 9, procedure: In which language(s) was the training conducted? Did the trainees have sufficient command of these, and were these the languages in which they would then explain the AIM-T to participants/patients? How did the authors ensure that the information was not lost in translation? | The training was conducted in French for Burundi, Cameroon and Central african republic, all raters being fluent in French, while the training in Iraq was provided in English, with Arabic translation to avoid misunderstanding.  French might not have been the language spoken to all patients. However, each item was described, and illustrated with videos during the training, to ensure a full understanding of each of the 12 activities by the raters. | The language used during the trainings has been added in the procedure section of the Methods, as well as mitigations for translation misunderstanding (i.e. use of videos). |
| - Page 13: please add some detail on most common injuries. | Thank you for identifying this gap. | The frequencies in the different injury location subgroups have been moved to the text, while additional information has been added in table 1. |
| Discussion:  - Language and (health) literacy issues: the authors do not mention anywhere in the manuscript o If they faced language barriers in the use of the BI and which language versions were used, respectively; o If they faced language barriers during trainings and application of the AIM-T and how to overcome these, see comment re page 9 | For BI, the French and Arabic validated versions were used, and revised following a linguistic validation process, instructed by BI license holders. Language and cultural barriers were identified during the linguistic validation in each of the four centres, highlighting the cultural sensitivity of asking self-care and bowel/bladder questions to patients, as well as the variability in performing some of the activities (such as transfer to toilet and eating).  Regarding the AIM-T, feedback was gathered and incorporated during the content validity testing (Gohy et al. 2022). Additionally, during this study, raters expressed the need to have illustrations for patients to understand better the activities to be performed. Drawings (S4 fig) were developed based on those feedbacks. | Differences in ways the different activities are understood by patients are now more clearly addressed in the fourth paragraph of the discussion. |
| o in Clinical implications: Training package / e-learning module sound very valuable, are these delivered in English, French, Arabic, local languages? Will they be available outside the INGO world? Please add | The e-learning is currently being developed in English, it will then be translated in French in the autumn and the Arabic translation is planned for 2024. Translations in local languages have not yet been considered. We have rather prioritised languages spoken most commonly by the healthcare professionals themselves.  All materials produced throughout the research are open access. The e-learning will be published on Tembo (MSF learning platform) and Disaster Ready, both of which are open to all for free. | Additional information on the e-learning languages has been added in the clinical implication paragraph. |
| - It seems as if AIM-T was developed for settings where INGOs lead or support projects. A measure like the AIM-T is crucial for any fragile settings and it should not be applied only in the presence of INGOs. The question is how it can be of help for local structures in fragile systems and how it can be transferred to local knowledge to enhance standards and practice around outcome measurement. The discussion/ limitations sections should include some reflections on this. | Thank you for pointing out this need for clarification.  The AIM-T was developed for humanitarian settings, where needs exceed coping capacity, which requires additional resources regardless of its origin (i.e. international or national / regional), therefore not specifically for INGOs.  All training materials will soon be available as open access, to facilitate their uptake.  Local stakeholders have been involved throughout the research, and local workshops have been organized in the study sites, sharing results with local stakeholders. | This is now clarified in the limitations and clinical implications and future research sections of the discussion, also highlighting the importance of a stronger health information system within those settings. |
| - Clinical implications and future research : o “Although feedback from the field teams is supportive, it is still to be studied whether the AIM-T actually promotes early mobilization at patient and clinician level.” – Although early mobilization should indeed be promoted, I am not convinced the development of a measure will or should have that purpose; A measure designed for humanitarian settings should allow outcomes to be validly and routinely measured - a well known barrier- to determine the impact of early mobilisation, prevention of permanent impairment etc | Thank you for the interesting comment, and we understand the reviewer’s concern.  However, as expressed in an earlier comment, we believe that in humanitarian settings, where the importance of early mobilization is not necessarily ubiquitously recognized, the considered use of monitoring systems can also affect clinical practice. By including mobility aspects in the monitoring tool, we believe that clinicians are more likely to resort to mobilization, on the one hand simply to be able to complete the monitoring activities, and on the other hand by recognizing the importance of mobility due to its inclusion in the monitoring and evaluation process.  As highlighted, this study does not measure this potential impact, and future studies would be required to address this hypothesis. | This aspect has been rephrased. |
| o the overall research project includes various steps in validation and use of the AIM-T, which is excellent, and the authors refer to a subsequently planned study. They are hereby addressing a hugely underresearched subject. However, I would also like to see some reflection on future research needs in the sector and in humanitarian settings, such as studies on overall rehabilitation impact measurement, rehabilitation outcome measurement development / cultural + language validation, and more. | Thank you for highlighting this important gap | The future research section has been revised. |
| - References: 38 incomplete | Thank you for identifying this typo. | We have now revised the reference 38 (now 39). |
| - S4_Fig: Is there a reason why the activity to get up from the floor into standing and back is not part of the AIM-T? In many humanitarian contexts, this is a typical daily function. | The content validity of the AIM-T has been tested and described in another manuscript (Gohy et al. 2022).  The floor transfer is indeed key in many different settings, highlighted by others (Prakash et al. 2016). It is to an extent integrated in the kneeling item (where the patient needs to kneel down to floor and stand up again), while the core items (i.e. roll over, and sit up and remain seated) tackle the initial transfers needed to sit up from laying position. For the sake of brevity, we have therefore not added a specific “floor transfer” item, and such addition was also not prompted by study participants in the content validity study. | No change has been made. |
